# Managing Children’s Fears during the COVID-19 Pandemic: Strategies Adopted by Italian Caregivers

**DOI:** 10.3390/ijerph191811699

**Published:** 2022-09-16

**Authors:** Marta Landoni, Sergio A. Silverio, Chiara Ionio, Francesca Giordano

**Affiliations:** 1 CriDee Department of Psychology, Università Cattolica del Sacro Cuore, 20123 Milan, Italy; 2 Department of Women & Children’s Health, King’s College London, London SE1 7EH, UK; 3 RiRes Department of Psychology, Università Cattolica del Sacro Cuore, 20123 Milan, Italy

**Keywords:** fear, COVID-19, children, parents, coping strategies, family resilience, qualitative, content analysis

## Abstract

Background: Life-threatening events, such as the COVID-19 pandemic, may generate feelings of insecurity and fear in the affected population, particularly children. Parents’ ability to help children cope with negative emotions is essential during challenging periods. The current study aims to analyse the coping strategies adopted by Italian caregivers concerning their children’s fears about COVID-19. Method: An online survey was administered during the Italian lockdown to 649 parents of at least one child aged 5 to 17 years old. Respondents completed the survey for themselves and their children. In addition, a qualitative content analysis of the data from the open-ended question was conducted (N = 569; 87.9% women; M_Age_ = 45 years). Results: Several themes were identified. Firstly, families’ primary approach was ‘communication and meaning-making’. Secondly, another essential strategy was ‘the importance of safe space’, enabled by keeping routine in place and creating a loving and caring environment. Thirdly, other factors relevant to managing children’s fears were ‘adaptation’, ‘religion’, ‘a positive attitude’, and ‘humour and hope’. Finally, the last two strategies significant and valuable for Italian families were ‘flexibility’ and ‘maintaining virtual contacts. Conclusion: During the pandemic COVID-19, parents may have used various strategies to protect their children from stress. Future research could investigate single parents’ coping strategies explicitly developed during the COVID-19 lockdown.

## 1. Introduction

Italy was the first European country to be afflicted by the SARS-CoV-2 pandemic, a novel coronavirus (COVID-19) originating in the city of Wuhan in China’s Hubei province. The measures taken by the Italian Government to contain the spread of the virus were mainly based on quarantine and social distancing, with the approximate population of 60 million Italians being asked to stay at home from March 2020, initially for two months, but for various periods as each region suffered spikes in infection rates and infection-related mortality. Like many countries, the necessary restrictions on daily life caused dramatic economic, social, and psychological consequences [1,2]. 

Therefore, experts around the world were rightly concerned about the psychological impact of the pandemic and its associated restrictions on the global population [3] and especially the effects it would have on children [4,5]. School closures and a lack of outdoor activities drastically disrupted children’s daily routines during the COVID-19 outbreak. Attachment, inattention, and irritability were the most severe symptoms in children during the pandemic, according to a preliminary study in China [2,6].

Orgiles et al. [7] observed similarities in two European countries: Italy and Spain. They found a deterioration in children’s emotional state and behaviour, especially in assessing concentration problems, boredom, irritability, and loneliness.

Moreover, it has been found that children’s inadequate capacity to understand and process the events during and after the disaster made the young population even more vulnerable as they experienced anxiety and insecurity [8,9]. Indeed, reports suggest that children became increasingly concerned about COVID-19 and their health, friends, and family as the pandemic continued [10].

Morens, Folkers, and Fauci [11] (p. 715) noted that *“emerging infections remain among the greatest challenges to human survival.*” In particular, contagious diseases arouse anxiety because the infection is *“transmissible, imminent, and invisible”* [12] (p. 744). However, in the literature, stress and anxiety reactions are considered normal and potentially adaptive or protective responses to infectious diseases [13]. 

Schimmenti et al. [14] described four fears associated with COVID-19: fear of the body, fear of significant others, fear of action, and fear of knowledge. For children, fear of significant others was identified as the most important. Most childhood fears are short-lived; however, specific fears may persist during a pandemic as the risk dominates a significant portion of their lifetime, with the propensity to impair normal psycho-social and emotional functionality.

Research conducted during the previous contagious outbreaks showed that parents’ and children’s fears of infection were significantly correlated [15]. Specifically, parents’ fears were related to the risk of transmission to their children, which, in turn, was related to children’s fear of the disease. Thus, parents could significantly influence children’s well-being in a health crisis, as children’s anxieties begin in the family environment. Furthermore, in anxiety-management studies, the parents’ position is the most frequently studied factor in dealing with children’s specific concerns [16,17]. Consequently, parents’ ability to effectively manage their worries and deal with negative emotions is crucial in difficult times such as the COVID-19 pandemic [10]. 

We can assume that many children will need additional support and reassurance from their parents during and after the COVID-19 pandemic and adequate and straightforward information to understand events. Thus, finding ways to lessen dysfunction and improve functional coping is crucial for children’s immediate and prospective development. While there have been a few studies on the relationship between children’s coping strategies and their recovery from a disaster [18], no-one has looked at how children develop suitable coping mechanisms for a pandemic [5]. Coping socialisation is a term used in the non-catastrophic events literature to describe the parental goals and practices that influence children’s use of coping strategies to deal with stressful events considered to be too big for the child to handle by either the parent or the child themselves [18,19,20]. We do not know much about how parents model coping behaviour or socialise their children’s usage of coping methods in the aftermath of pandemics. Therefore, research is necessary because neither the developmental model of children’s age-related coping processes nor models of coping socialisation have been applied in the context of such stressors [18]. Given the importance of parental influence on developing their children’s competence in health and safety [21], parents likely play a role in helping their children learn to cope effectively with pandemics.

Based on this theorisation, our research aims to explore the coping strategies parents of Italian children used to alleviate their children’s anxieties during and after the COVID-19 pandemic.

## 2. Materials and Methods

### 2.1. Design and Procedure

The present study is part of a larger research project entitled *‘Strategies for building resilience in families dealing with the COVID-19 pandemic’.*

The study protocols were approved by the Universidad de Barcelona, specifically by the Department of Didactics and Educational Organisation, in collaboration with Professor Anna Fores Miravalles.

The online survey involved parents residing in Italy and was conducted between 21 April and 27 May 2020. Participants were asked to complete the study, in which they were asked to reflect on their behaviour, feelings, and thoughts, as well as those of their children in the current lockdown situation. 

After providing consent, participants answered demographic questions (e.g., age, education, etc.) and a series of psychometric assessments related to parental resilience and mental health, family resilience, and children’s emotional and behavioural difficulties (the results will be reported elsewhere). At the end of the survey, open-ended questions addressed various questions on resilience and the current pandemic situation. For this analysis, only information from the open-ended qualitative questions was used. Specifically, we focus on the question: *“How did you help your child cope with anxiety related to the current situation?”*

The survey took approximately 30 min to complete. After completing the study, parents received cards created by the first authors’ research institute with ten tips for building family resilience during disasters. 

### 2.2. Recruitment and Participants

A snowball sampling technique was employed, with the link to the survey disseminated via invitations sent by e-mail, WhatsApp, and social media (Facebook and Instagram). Inclusion criteria were ≥18 years of age; ability to provide informed consent; place of residence (Italy); and having custody of and/or living with their children. A convenience sample of Italian parents (N = 569; 87.9% women; M_Age_ = 45 years) was recruited for the study (see Table 1 for complete demographics); 39.40% of them experienced a total lockdown, 26.90 partial restrictions, 32.40% were out of town, and 1.30% of them were out of the region. In total, 73.6% were married, 13.9% were cohabiting at the time of the survey, 6.7% were widowed, 1.4% were divorced, and 1.5% were single. Most participants were from Lombardy (53.8%) and Sardinia (27.2%), and the rest were from all other Italian regions. In addition, 61% of the sample were full-time workers, 24.6% were part-time workers, and the rest were casual (4.60%) or unemployed (8.10%). Only 13.3% had been infected with COVID-19; 30.9% had relatives affected by the virus, and 8.8% had experienced the loss of a family member to coronavirus in 2020.

Before beginning the analysis, the data were sorted by the date received, starting with the oldest responses to assess temporal data saturation. Analysis revealed that the most recent answers did not raise new themes, but could be related to the themes raised by the older answers, thus allowing data collection to cease. 

A qualitative content analysis [22] was deemed the most suitable approach to illustrate the pandemic phenomenon, which had otherwise not been examined before and for which an established knowledge base was lacking. This methodology allows for the content of textual data to be identified through a systematic categorisation process, producing explicit themes in the text that can be interpreted. 

Open coding, classification, and abstraction were the three steps in the organising phase. One researcher delved into the data and tried to create new themes from the collected data in the organising phase. Then, they labelled the new categories which contained the themes of the group. In the coding phase, each transcript was read numerous times before being analysed to better understand the experiences and feelings of the study participants. Next, codes were organised during the categorisation phase. Finally, grouping related codes into larger clusters categorised the data to minimise the number of overall categories. The presentation of the results was the final step in the content analysis process. 

Coding this content was performed by two independent raters (ML and GM) who coded the written content separately and then compared their coding.

When different categories were identified, a comparison was made, which in most cases resulted in an agreement between the coders or a combination of the categories into a more general one. However, where agreement could not be reached, a third, more experienced researcher (FG) was involved in resolving the discrepancy. 

The final sample of this qualitative analysis included 563 participants out of a total of 569 as six responses were unattributable. Parent frequencies were calculated as the number of occurrences of a particular theme in the responses within the total number of themes observed. Inter-rater reliability was calculated and was approximately 88% as the number of matches between the independent raters divided by the total number of individually coded materials [23].

## 3. Results

All of the themes identified during the analysis of 569 responses are presented in Table 2. Out of all the responses, some (n = 71; 13%) reported no fears about the coronavirus. The remaining answers were sorted into five coding clusters. 

### 3.1. Communication

As shown in the table, the essential strategy used by parents was meaning-making and communication (n = 316; 55.53%). In addition, another strategy employed was transparent communication which emphasises the importance of social restrictions (n = 51; 8.96%). The last two methods related to an area of communication were using simple language (n = 33; 5.79%) and engaging children’s feelings and opinions (n = 26; 4.56%). 

### 3.2. Information Sharing and Censoring

Another critical macro area was information sharing, which included two strategies: informing children (n = 50; 8.38%) and limiting contact with mass media (n = 20; 3.51%). Indeed, media (e.g., TV or radio) and social media (e.g., Internet-based social networks) significantly influence children’s moods, emotions, and behaviour. 

### 3.3. Emotion Regulation

Regarding emotions, different strategies were used. The most common was reassuring children about the negative effects of COVID-19 and managing their emotions (n = 50; 8.38%). This was followed by other coping strategies: parents’ ability to share their feelings (n = 15; 2.63%), maintain positivity (n = 15; 2.63%), and the ability to positively restructure emotions (n = 8; 1.4%), whilst reframing the situation (n = 23; 4.04%), using techniques of humour (n = 5; 0.87%), and being hopeful (n = 10; 1.75%).

### 3.4. Focus on Family

Family cohesion and coping behaviours were two other macro domains discovered. In terms of family cohesion, four strategies were most prevalent among parents: proximity to each other (n = 19; 3.4%), physical contact (n = 16; 3.40%), virtual contact with wider friends and family (n = 11; 1.93%), and providing security and a safe place (n = 9; 1.6%). 

### 3.5. Routines and Distraction Techniques

Regarding coping behaviours for this cluster, two strategies were found as distraction techniques: engaging children in leisure activities (n = 20; 3.5%) and the importance of outdoor spaces (n = 1; 0.1%). Two were identified as being linked: keeping a routine (n = 5; 0.87%) and maintaining a religious connection (n = 2; 0.3%). Together, these four mechanisms signalled an ability to adapt (n = 6; 1.06%).

**Table 2:** Clusters and coded themes identified from the qualitative analysis provided examples of statements and percentages.

Coding Cluster	Coded Theme	Description of Theme	Example Quotations	Frequency of Occurrence	Percentage Cover
**Communication**	Meaning-making and communication	Give children an explanation of the pandemic and talk with them about it	*“Explaining to her what the virus was and why we needed to stay home”.*	316	55.53%
	Communication about the restrictions	Communication about the importance of adopting restriction behaviours	*“Reassuring her that containment measures are there to prevent contagions so that we can return to safer health outcomes.”*	51	8.96%
	Use of straightforward language	Use of simple language to communicate with children	*“We explained to them what the situation is and what measures have been taken to avoid the dangers of this virus, obviously in a language suitable for two children of 4 and 6 years old.”*	33	5.79%
	Give voice to children’s emotions and opinions	Help children understand how they feel and share their feelings	*“Making him tell and describe his feelings in full freedom.”*	26	4.56%
**Information Sharing and Censoring**	Keeping children informed	Use of video, books, mass media, and school lessons to inform children	*“We explained to her with the help of a fable created by the teachers the coronavirus and its consequences on our lives.”*	50	8.38%
	Limiting mass-media exposure	Using a filter to the information provided by mass media	*“I didn’t allow him to see too much news on TV.”*	20	3.51%
**Emotion Regulation**	Managing emotions	Emotional reassurance for children	*“Trying to reassure.”*	50	8.38%
	Reframe	Reframe the situation. Ability to switch their point of view in a positive way	*“Living this experience for its positive sides: we were able to spend time all together as on holiday time.”*	23	4.04%
	Emotional coping	Sharing emotions and maintaining calm behaviour	*“Sharing fears.”*	15	2.63%
	Positive attitude	Maintaining a positive attitude	*“Explaining that this is a situation that will have an end anyway.”*	15	2.63%
	Regulation of emotions	Positive restructuring of emotions	*“Fortunately, since we were all physically well, it was easy to explain to him that fear does not always correspond to a necessarily negative consequence… by being careful, we can continue to be well.”*	8	1.40%
	Hope	Re-envisioning the future	*“Keeping hope alive in the future”*	10	1.75%
	Humour	Use of humour	*“I was ironic. By saying that 12-year olds don’t die and that it was us as parents who were eventually destined. Using macabre humour.”*	5	0.87%
**Focus on Family**	Nuclear family Unit	Being closed to each other	*“Stay close to him.”*	19	3.40%
	Affection	Physical contact and demonstration of love	*“Hugging each other and letting him know that mom and dad are with him.”*	16	3.40%
	Technology	Maintaining virtual contacts to connect each other	*“Trying to create opportunities for virtual closeness with loved ones (grandparents, friends, classmates).”*	11	1.93%
	Feeling safe	Creation of a safe place	*“He has always felt safe in the family.”*	9	1.60%
**Routines and Distraction Techniques**	Leisure activities	Engaged children in leisure activities	*“With drawings and games. Impersonating the virus with puppets.”*	20	3.55%
	Routine	Trying to re-create an everyday life routine	*“Trying to recreate normality.”*	5	0.87%
	Adaptation	Capacity to adjust themselves in a new situation	*“Easily gets used to new conditions.”*	6	1.06%
	Religion	Having faith	*“God has a good plan for us.”*	2	0.30%
	Nature	Importance of nature and outdoor spaces	*“Trying to live the quarantine also ensuring moments outdoors almost daily.”*	1	0.17%
**No Concerns**	Fears not related to COVID-19	Absence of fears or fears correlated to other topics	*“She did not have concerns about the current coronavirus situation, but more about the internal family dynamics.”*	71	12.47%

## 4. Discussion

Life-threatening events, such as a pandemic, may generate feelings of insecurity, fear, desperation, stress, and anxiety [24,25] in the affected population and, particularly, in children [26,27,28]. It is likely that families during the COVID-19 pandemic find within themselves a source of resilience to cope with multiple stresses, adapt to devastating dislocations, strengthen important bonds, endure uncertainty, and manage future challenges [5]. In particular, studies conducted on children exposed to adversities have shown the need for mutual support, comfort, and protection when helplessness and confusion are widespread [29,30,31,32]. Children learn coping strategies either directly, e.g., when parents encourage their children to use specific techniques to cope with stressors or their reactions to them [33], or indirectly, e.g., when children observe their parents’ emotional, cognitive, and behavioural strategies for coping with stressful events [18,34]. 

Children’s ability to respond adaptively to natural disasters is influenced by their parents’ coping (functional or dysfunctional) with stressful situations and their ability to adapt their own coping to that of their parents [35].

Based on this theoretical framework, we aimed to explore the strategies and resources Italian families and parents use to help children cope with their fears and anxieties. Many previous studies lacked a clear theoretical framework which could provide an overview of coping strategies during pandemics. Research which has looked at families during a pandemic has mostly focused on the risks they face [36] rather than their actual coping methods; therefore, this work makes an original contribution to the literature, as studies on family coping strategies during the current pandemic remain scarce. 

Moreover, coping strategies play an essential role in the conceptual framework for understanding mental health problems following trauma. However, in these models, coping has been examined as a post-traumatic component that influences the development of post-traumatic reactions [37]. The current study examined how families cope with the frightening event of COVID-19. Long-term treatment and preventive early intervention strategies could benefit from a better understanding of the full range of managing experiences both during the acute crisis and post-trauma period [37]. Coping mechanisms which may be effective after trauma may not be effective when trauma is ongoing. Research also underscores the importance of viewing coping from a developmental perspective [37]. Although many of the children’s coping mechanisms are similar to those described in the adult literature, their experiences demonstrate how essential adults are to young people in assessing danger, controlling their emotions, and feeling comfortable [37]. From a clinical perspective, this would imply that children of any age who are cut off from protective adult figures during trauma feel more powerless and have fewer coping mechanisms available compared to adults. Whether the presence of adults during trauma leads to more positive coping mechanisms and fewer long-term health effects should be the subject of further investigation [37]. Given the likelihood that humanity will be affected by additional pandemics in the future, understanding how families support themselves in this scenario to protect children is an important goal for academics to pursue at this time. 

In our study, we first noted that the direct approach used by Italian families to help children cope with their fears is meaning-making, which is consistent with other literature on traumatic events [38]. According to Walsh, meaning-making is a vital family belief system strategy. Meaning-making could be considered an essential healing process in response to traumatic events by providing emotional support to children [27,31]. However, as described in the previous literature, it may have been difficult for parents to understand what was happening in this pandemic if they had no prior experience. As explained in previous studies, parents had to gradually make sense of the situation, what could be known, and the uncertainty that remained as they grappled with the consequences [38]. According to the literature, the meaning-making processes in families involve joint attempts to make sense of the situation by making it more bearable for the children and integrating it into their personal and relational life trajectories [38].

Therefore, the processes of meaning-making in families involve joint attempts to make sense of the situation by making it more bearable for the children and integrating it into the children’s personal and relational life trajectories, as pointed out in our study by one participant: *“I have emphasized that it is not necessary to worry about things that are not up to us, such as the course of the pandemic, but about things where our action help to change the situation, such as protecting ourselves with masks and maintaining social distancing. This attitude must be implemented for every difficulty in life to avoid unnecessary stress.”*

Our study has also used several communication strategies between parents and children. First, parents adopt language and communication to the child’s age by giving clear information. Boss [39], for example, highlighted how ambiguity in crises could block communication between members and their closeness. Sometimes, however, even well-functioning families avoid painful or frightening conversations to protect each other from worry until there is certainty about such situations. Nonetheless, fears of unspoken words can cause widespread anxiety and often become somatised or lead to behavioural problems, especially in children [40]. Therefore, parents or caregivers should intervene by informing children whenever the situation develops, discussing it openly, and addressing concerns or doubts. In such cases, guidance on age-appropriate forms of communication may be needed, and considerations may be made as children have grown older to allow for better understanding or to address new concerns [41].

Many parents also stressed the need to provide age-appropriate amounts of information to help their children. Parents stated that they presented clarifications of catastrophe occurrences, downplayed the danger levels of disaster cues, and did not discuss the probable repercussion with pre-school or early primary school-aged children. Children and adolescents received factual information without over-dramatizing the severity of the potential consequences to life or property, and some parents engaged them in supporting others through recovery activities [18]. In addition, the literature has shown how unclear or mixed messages during major disasters increase anxiety levels and block understanding of what is happening, how to get out of the situation, and what to expect in the immediate future. Instead, families who clarify and share essential information about their situation can give children new meaning to the situation they are experiencing [42]. Our study has shown that a way to provide children with information may be with age-appropriate materials (e.g., videos, etc.): *“We explained to her right away with the help of a fable created by the teachers about the coronavirus and its consequences on our lives.”*

In other studies, teachers or psychologists and health care workers were instrumental in providing these materials to families. Saxena et al. [43] explained the crucial role of teachers in conveying information through various techniques such as listening, describing, analyzing, demonstrating, and emphasizing.

Another way to convey information found in our study is by limiting children’s media exposure. Indeed, children and adults respond differently to information related to the current situation, and their responses can be influenced by their level of media exposure [44]. Media such as TV, radio, and social media have been shown to have a significant impact on children’s mood, emotions, and behavior [45]. The literature on coping strategies and COVID-19 distinguishes between problem-oriented and emotion-oriented coping methods. The most common problem-oriented strategies included listening to experts, following their advice, and carefully considering what to do next [46]. Parents must limit children’s exposure to information [47,48]. This action could help parents identify and monitor children’s knowledge and offer information more appropriate to their developmental stage [49]. 

It is essential to give voice to children and their thoughts or emotions, and parents in this study created spaces and conditions in which they could listen to children’s stories. Children learn to name their feelings by observing those around them. Therefore, parents should support this learning by engaging them in warm and respectful conversations to help their children identify and evaluate their feelings. 

In our study, parents provided informational and emotional support, reframed potentially negative emotions and adapted their parenting style to the children’s varied responses to the crisis [18]. According to Miller et al. [18], as a result, one of the most important roles for parents in assisting children in coping with and adapting to natural catastrophes is to effectively model self-regulation of negative emotional states and help their children in learning skills for controlling negative arousal.

In our study, according to previous studies, three main ways of emotional coping were chosen by Italian parents once children’s emotions had been heard: calming, sharing feelings, and positive restructuring of emotions. By sharing their feelings about the pandemic, children and parents can feel uplifted and feel less alone in this critical situation. Therefore, it becomes essential that adults identify, recognise, label, and self-regulate the wide range of emotions experienced due to the pandemic to help their children do the same [49]. However, little is known about how these forms of support may influence children’s acquisition of strategies to regulate their affective state in stressful situations, so the relative contribution of parents’ emotional support strategies to children’s later adjustment remains an important area of research [50]. Furthermore, young children require parents who can provide emotional stability and dependable care. In our study, providing youngsters with a secure environment and a sense of security was linked to prosocial behaviour and routines [18].

A life-threatening event such as a virus can trigger feelings of fear or despair, making children feel insecure. Parents should make sure that children know that they are safe and that virus is only a temporary event. During COVID-19, the daily routine is lost, and children find themselves with similar days. Children may fear this new normal because they miss their ‘old life’ and daily routine before COVID-19. Therefore, parents need to help their children maintain the way at home as much as possible by keeping the children’s activities and hobbies alive. Indeed, studies have shown how parents’ empathetic care and basic daily routines can provide young children with security and a sense of normalcy [50]. For older children and young people, the challenge can be far greater as their lives are embedded with much more childcare, school, friendships, and leisure activities, which is much more difficult to restore [51]. In our study, parents’ strategies for maintaining routines and creating a loving and caring environment were based on: *“Trying to recreate normality.”*

Further, our study also highlighted other individual strategies such as adjustment, reframing, religion, positive attitude, humour, and hope. Individuals with a high level of hope during times of fear are also known to have a high level of resilience and well-being [52]. It is common to experience discouraging difficulties in confronting significant challenges with COVID-19. The mutual encouragement of family members could strengthen ongoing efforts to take the initiative and persevere. Indeed, during a tough time, affirming individual and family strengths can counter a lack of agency; disappointment; fear; a lack of trust; and foster a ‘can-do’ attitude [52].

Further, it could be that parents and children, because they feel threatened, might tend to see everything negative. Although this could be considered a ‘normal’ reaction, parents should try to reduce it by using positive thinking so that children can model their behaviours. Previous research has documented the positive effects of faith in times of crisis, belief in a higher power, prayer, and meditation practices [53]. The last two strategies in our article are significant and valuable for Italian families to help children deal with their fears: flexibility and maintaining virtual contacts. Flexibility is required to change parts and familiarise one with transformed conditions and unexpected tasks. At the same time, it is essential to maintain contact with immediate and extended family. Indeed, social isolation does not mean parents and children cannot use technology to interact with their social support networks. On the contrary, parents should create opportunities for children to keep in contact with family members and friends.

## 5. Conclusions

Masten and Obradovic [54] (p.9) affirm that during a disaster, “*families often infect each other before a person is diagnosed, and they also infect each other with fear*”. During the COVID-19 pandemic, children were exposed to a large amount of information which may have caused high levels of stress and anxiety in them and their caregivers. Parents may have used different strategies to protect their children from stress: some may have avoided talking about complicated feelings and events, while others may have given explanations to their children to ensure they did not feel unnecessarily anxious or guilty. 

The aim of this study was to qualitatively investigate the coping strategies of Italian parents in dealing with their children’s fears during the COVID-19 pandemic. Although this study provides new insights into the strategies used to cope with their children’s fears during a traumatic event such as a pandemic, it has some limitations. It is worth noting that our study mainly examined families with two parents. Although the results highlight the importance of coping strategies at the relationship level, single parents may not have access to some of the strategies used in two-parent families, such as responsibility sharing. Future research could further examine the strategies used with particular attention to family composition (single parent or family) and other demographic variables. In fact, the researchers aimed for a sample that was reasonably representative of different demographic groups. However, sample selection depended on the willingness of families to participate in the study. As a result, these demographic characteristics were not evenly represented in the sample.

Psychometric assessments of children’s and their parents’ mental health (e.g., anxiety) may also prove useful. Future research should address additional strategies that parents might use to quantitatively understand their children’s anxiety about COVID-19. Further research is needed to identify helpful strategies and develop materials to assist parents during such a difficult time.

## Figures and Tables

**Table 1:** Demographics of the sample, described for variables and percentages.

Gender		Region		Child’s Age	
Male	12.10%	Lombardy	53.70%	0–4 Years	24.50%
Female	87.90%	Sardinia	27.30%	5–12 Years	38.00%
		Piemonte	6.70%	13–17 years	37.30%
**Marital Status**		Sicily	2.60%		
Married	73.60%	Emilia-Romagna	1.60%	**Work**	
Common law marriage	13.90%	Tuscany	1.60%	Full time workers	61.00%
Widow	6.70%	Abruzzo	1.20%	Part-time workers	24.60%
Single	4.40%	Lazio	1.10%	Unemployed	8.10%
Divorced	1.40%	Liguria	1.10%	Day laborer	4.60%
		Marche	1.10%	Student	1.80%
		Veneto	0.90%		
		Campania	0.50%		
		Puglia	0.20%		
		Calabria	0.20%		
		Valle D’Aosta	0.20%		

## Data Availability

Given the sensitive nature of the data produced by this study, the survey responses will not be made publicly accessible.
